# Supplementary material for: The Psychological Impact of Coronavirus Disease 2019 on Patients Attending a Tertiary Healthcare Facility in Pakistan: A Cross-Sectional Study
Source: Healthcare (Basel). 2022 Jun 5;10(6):1049. doi: 10.3390/healthcare10061049 (PMC9223015; doi:10.3390/healthcare10061049)
Supplement: Supplementary file 1 [file healthcare-10-01049-s001.zip › healthcare-1726210-supplementary.pdf]

DASS-21 and PCL-5 in Treated COVID-19 Patients:

This is a questionnaire to measure Depression, Anxiety and PTSD in treated COVID-19 patients and Healthcare Workers.

صحت یاب کرونا وائرس کے مریضوں اور ہیلتھ کیئر ورکرز میں افسردگی ، بے چینی اور تناؤ کی پیمائش کرنے کے لئے سوالنامہ.

Name/ نام : ______________________________

Age/عمر: ________________________________

Gender/جنس: ____________________________

Education/تعلیم: __________________________

Occupation/پیشہ: _________________________

Healthcare Worker/ہیلتھ ورکر(yes/no): ________

History of interaction with COVID-19 patient in hospital as Frontline worker/ہسپتال میں کرونا وائرس کے مریضوں کا علاج کیا فرنٹ لائن پر: ____________________________

Severity of Disease/بیماری کی شدت(Mild/Moderate/Severe/Critical): ______________

Current Condition/موجودہ حالت (same as before illness/worse/better) : ______________

Informed Consent/مطلع شدہ اجازت نامہ:

I hereby give my consent for clinical information related to me to be reported for research purposes.
I understand that my name and identity will be concealed.
Once signed I cannot revoke consent.

____
(Patient's Signatures/دستخط)

DASS-21 Questionnaire: (Encircle the right option/صحیح انتخاب پر دائرہ لگائیں)

Sr. No.	Characteristics/ خصوصیات	Never کبھی نہیں (0)	Some times کبھی کبھار (1)	Often اکثر اوقات (2)	Always ہر وقت (3)
1.	**I found it hard to wind down.**<br>میرے لئے پر سکون رہنا مشکل ہو رہا تھا.	0	1	2	3
2.	**I was aware of dryness of my mouth.**<br>میں اپنے منہ کے سوکھے پن سے واقف تھا.	0	1	2	3
3.	**I couldn't seem to experience any positive feeling at all.**<br>مجھے کسی قسم کے مثبت جذبات محسوس نہیں ہوئے.	0	1	2	3
4.	**I experienced breathing difficulty (e.g. excessively rapid breathing, breathlessness in the absence of physical exertion).**<br>مجھے سانس لینے میں دشواری کا سامنا کرنا پڑا (بغیر کسی قسم کی مشقت کے).	0	1	2	3
5.	**I found it difficult to work up the initiative to do things.**<br>مجھے کسی کام کے کرنے کے لئے آغاز کرنا مشکل محسوس ہوتا رہا ہے.	0	1	2	3
6.	**I tended to over-react to situations.**<br>میں نے بعض حالات میں غیر ضروری ردعمل کا اظہار کیا.	0	1	2	3
7.	**I experienced trembling (e.g. in the hands).**<br>مجھے کپکپاہٹ محسوس ہوتی رہی ہے (مثلاً ہاتھوں میں).	0	1	2	3
8.	**I felt that I was using a lot of nervous energy.**<br>میں نے محسوس کیا کہ میں بہت زیادہ ذہنی توانائی استعمال کر رہا/رہی ہوں.	0	1	2	3
9.	**I was worried about situations in which I might panic and make a fool of myself.**<br>میں ایسے حالات سے گھبراتا رہا جن میں میری بے چینی بڑھنے اور احمق بننے کا خدشہ تھا.	0	1	2	3

10.	**I felt that I had nothing to look forward to.**<br>میں اپنا مستقبل تاریک محسوس کرتا رہ /کرتی رہی.	0	1	2	3
11.	**I found myself getting agitated.**<br>مجھے اپنے آپ میں چڑچڑا پن محسوس ہوتا رہا.	0	1	2	3
12.	**I found it difficult to relax.**<br>میں ذہنی طور پر بے سکونی محسوس کرتا رہا.	0	1	2	3
13.	**I felt down-hearted and blue.**<br>میں اداسی محسوس کرتا رہا /رہی.	0	1	2	3
14.	**I was intolerant of anything that kept me from getting on with what I was doing.**<br>میرے لئے اس چیز کو برداشت کرنا مشکل ہو رہا تھا جو میرے کام میں رکاوٹ پیدا کرے.	0	1	2	3
15.	**I felt I was close to panic.**<br>مجھے محسوس ہوتا تھا جیسے مجھے دورہ پڑنے لگا ہے.	0	1	2	3
16.	**I was unable to become enthusiastic about anything.**<br>مجھے کسی بھی کام میں دلچسپی نہیں رہی.	0	1	2	3
17.	**I felt I wasn't worth much as a person.**<br>مجھے محسوس ہوتا رہا کہ میں کسی قابل نہیں ہوں.	0	1	2	3
18.	**I felt that I was rather touchy.**<br>مجھے محسوس ہوا کہ میں بہت جزباتی ہو جاتا ہوں.	0	1	2	3
19.	**I was aware of the action of my heart in the absence of physical exertion (e.g. sense of heart rate increase, heart missing a beat).**<br>مجھے بلا وجہ، بغیر کسی جسمانی مشقت کے دل کی دھڑکن تیز محسوس ہوتی رہی.	0	1	2	3

20.	**I felt scared without any good reason.**<br>میں بغیر کسی وجہ کے خوفزدہ محسوس کرتا رہا /رہی.	0	1	2	3
21.	**I felt that life was meaningless.**<br>مجھے یہ احساس ہوتا رہا کہ زندگی بے معنی ہے.	0	1	2	3

PCL-5 Questionnaire: (Encircle the right option/صحیح انتخاب پر دائرہ لگائیں)

**Sr. No.**	**In the past month, how much you were bothered by:**<br>پچھلے مہینے میں، آپ ان باتوں سے کتنے پریشان ہوئے تھے:	**Not at all/**<br>بالکل نہیں<br>**(0)**	**A little bit/**<br>تھوڑا بہت<br>**(1)**	**Moderately/**<br>کسی حد تک<br>**(2)**	**Quite a bit/**<br>کافی حد تک<br>**(3)**	**Extremely/**<br>بے حد<br>**(4)**
**1.**	**Repeated, disturbing, and unwanted memories of the stressful experience?**<br>پریشان کن ، اور ذہنی دباؤ والے تجربے کی ناپسندیدہ یادیں بار بار آنا؟	**0**	**1**	**2**	**3**	**4**
**2.**	**Repeated, disturbing dreams of the stressful experience?**<br>ذہنی دباؤ والے تجربے کے بار بار ، پریشان کن خواب آنا؟	**0**	**1**	**2**	**3**	**4**
**3.**	**Suddenly feeling or acting as if the stressful experience were actually happening again (as if you were actually back there reliving it)?**<br>اچانک محسوس کرنا یا عمل کرنا جیسے ذہنی دباؤ والا تجربہ دوبارہ ہو رہا ہو (گویا واقعتا آپ واپس اسی کیفیت میں مبتلا ہو رہے ہیں) ؟	**0**	**1**	**2**	**3**	**4**
**4.**	**Feeling very upset when something reminded you of the stressful experience?**<br>جب آپ کو کوئی بات اس پریشان کن تجربے کی یاد دلوائے تو آپ بہت پریشانی محسوس کر تے ہوں؟	**0**	**1**	**2**	**3**	**4**
**5.**	**Having strong physical reactions when something reminded you of the stressful experience (for example, heart pounding, trouble breathing, sweating)?**<br>جب آپ کو کوئی بات اس ذہنی دباؤ والے وقت کی یاد دلاتی ہے تو آپ کو سخت جسمانی ردعمل کا سامنا کرنا پڑتا ہے (مثال کے طور پر ، دل کا دھڑکنا ، سانس لینے میں دشواری ، پسینہ آنا) ؟	**0**	**1**	**2**	**3**	**4**
**6.**	**Avoiding memories, thoughts, or feelings related to the stressful experience?**<br>ذہنی دباؤ والے تجربے سے متعلق یادوں ، خیالات ، یا	**0**	**1**	**2**	**3**	**4**

	احساسات سے بچنا؟					
7.	**Avoiding external reminders of the stressful experience (for example, people, places, conversations, activities, objects, or situations)?**<br>پریشان کن تجربے کی بیرونی یاد دہانیوں سے بچنا (مثال کے طور پر لوگوں ، مقامات ، گفتگو ، سرگرمیوں ، اشیاء یا حالات وغیرہ سے)؟	**0**	**1**	**2**	**3**	**4**
8.	**Trouble remembering important parts of the stressful experience?**<br>دباؤ والے تجربے کے اہم حصوں کو یاد رکھنے میں پریشانی کا سامنا کرنا؟	**0**	**1**	**2**	**3**	**4**
9.	**Having strong negative beliefs about yourself, other people, or the world (for example, having thoughts such as: I am bad, there is something seriously wrong with me, no one can be trusted, the world is completely dangerous)?**<br>اپنے ، دوسرے لوگوں ، یا دنیا کے بارے میں سخت منفی عقائد رکھنے (مثلا،: ایسے خیالات رکھنا جیسے: میں برا ہوں ، میرے ساتھ کوئی سنجیدہ مسئلہ ہے ، کسی پر اعتبار نہیں کیا جاسکتا ، پوری دنیا خطرناک ہے)؟	**0**	**1**	**2**	**3**	**4**
10.	**Blaming yourself or someone else for the stressful experience or what happened after it?**<br>پریشان کن تجربے کے لئے اپنے آپ کو یا کسی اور کو مورد الزام ٹھہرانا یا اس کے بعد جو کچھ بھی ہوا؟	**0**	**1**	**2**	**3**	**4**
11.	**Having strong negative feelings such as fear, horror, anger, guilt, or shame?**<br>سخت منفی جذبات جیسے خوف ، وحشت ، غصہ ، جرم یا شرم کا محسوس ہونا؟	**0**	**1**	**2**	**3**	**4**
12.	**Loss of interest in activities that you used to enjoy?**<br>جن سرگرمیوں سے آپ لطف اندوز ہوتے تھے ان میں دلچسپی ختم ہونا؟	**0**	**1**	**2**	**3**	**4**
13.	**Feeling distant or cut off from other people?**<br>دوسرے لوگوں سے دور ہونا یا منقطع محسوس کرنا؟	**0**	**1**	**2**	**3**	**4**

14.	**Trouble experiencing positive feelings (for example, being unable to feel happiness or have loving feelings for people close to you)?**<br>مثبت احساسات کے محسوس کرنے میں مشکل ہونا (مثال کے طور پر ، آپ کے قریبی لوگوں کے لئے خوشی محسوس کرنے یا محبت کے جذبات رکھنے سے قاصر ہونا)؟	**0**	**1**	**2**	**3**	**4**
15.	**Irritable behavior, angry outbursts, or acting aggressively?**<br>چڑچڑا پن ، ناراض ہو جانا ، یا جارحانہ انداز میں عمل کرنا؟	**0**	**1**	**2**	**3**	**4**
16.	**Taking too many risks or doing things that could cause you harm?**<br>بہت زیادہ خطرات مول لینا یا ایسی چیزیں کرنا جو آپ کو نقصان پہنچا سکتی ہوں؟	**0**	**1**	**2**	**3**	**4**
17.	**Being "superalert" or watchful or on guard?**<br>بہت زیادہ چوکنے، بہت زیادہ ہوشیار اور مستعد رہنا؟	**0**	**1**	**2**	**3**	**4**
18.	**Feeling jumpy or easily startled?**<br>بے صبری محسوس ہونا یا آسانی سے چونک جانا؟	**0**	**1**	**2**	**3**	**4**
19.	**Having difficulty concentrating?**<br>دھیان دینے میں دشواری ہونا؟	**0**	**1**	**2**	**3**	**4**

20.	**Trouble falling or staying asleep?**<br>سونے میں یا سوتے رہنے میں دقت محسوس ہونا؟	**0**	**1**	**2**	**3**	**4**
